# Comparison of Conventional Methods for Bowel Length Measurement in Laparoscopic Surgery to a Novel Computer-Assisted 3D Measurement System

**DOI:** 10.1007/s11695-021-05620-6

**Published:** 2021-07-30

**Authors:** Martin Wagner, Benjamin F. B. Mayer, Sebastian Bodenstedt, Karl-Friedrich Kowalewski, Felix Nickel, Stefanie Speidel, Lars Fischer, Hannes G. Kenngott, Beat-Peter Müller-Stich

**Affiliations:** 1 Department of General, Visceral and Transplantation Surgery, Heidelberg University Hospital, Im Neuenheimer Feld 420, 69120 Heidelberg, Germany; 2 Division of Translational Surgical Oncology, National Center for Tumor Diseases, Partner-Site Dresden, Fetscherstraße 74, 01307 Dresden, Germany; 3 Department of Urology and Urological Surgery, University Medical Center Mannheim, Heidelberg University, Theodor-Kutzer-Ufer, 68167 Mannheim, Germany; 4 Department for General and Visceral Surgery, Hospital Mittelbaden, Balger Str. 50, 76532 Baden-Baden, Germany

**Keywords:** Bowel length measurement, Quantitative laparoscopy, Metabolic surgery, Computer-assisted surgery, Stereo endoscopy

## Abstract

**Purpose:**

Accurate laparoscopic bowel length measurement (LBLM), which is used primarily in metabolic surgery, remains a challenge. This study aims to three conventional methods for LBLM, namely using visual judgment (VJ), instrument markings (IM), or premeasured tape (PT) to a novel computer-assisted 3D measurement system (BMS).

**Materials and Methods:**

LBLM methods were compared using a 3D laparoscope on bowel phantoms regarding accuracy (relative error in percent, %), time in seconds (s), and number of bowel grasps. Seventy centimeters were measured seven times. As a control, the first, third, fifth, and seventh measurements were performed with VJ. The interventions IM, PT, and BMS were performed following a randomized order as the second, fourth, and sixth measurements.

**Results:**

In total, 63 people participated. BMS showed better accuracy (2.1±3.7%) compared to VJ (8.7±13.7%, *p*=0.001), PT (4.3±6.8%, *p*=0.002), and IM (11±15.3%, *p*<0.001). Participants performed LBLM in a similar amount of time with BMS (175.7±59.7s) and PT (166.5±63.6s, *p*=0.35), but VJ (64.0±24.0s, *p*<0.001) and IM (144.9±55.4s, *p*=0.002) were faster. Number of bowel grasps as a measure for the risk of bowel lesions was similar for BMS (15.8±3.0) and PT (15.9±4.6, *p*=0.861), whereas VJ required less (14.1±3.4, *p*=0.004) and IM required more than BMS (22.2±6.9, *p*<0.001).

**Conclusions:**

PT had higher accuracy than VJ and IM, and lower number of bowel grasps than IM. BMS shows great potential for more reliable LBLM. Until BMS is available in clinical routine, PT should be preferred for LBLM.

**Graphical abstract:**

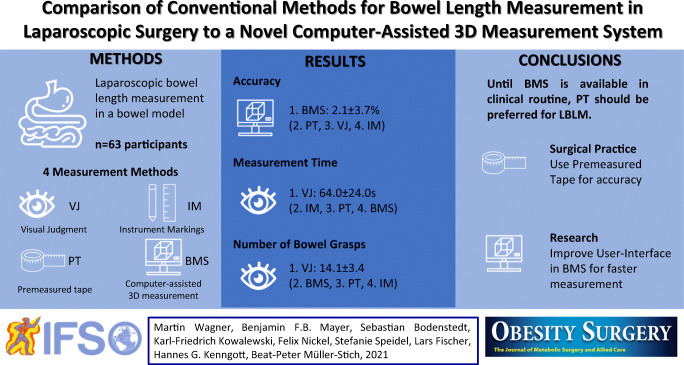

**Supplementary Information:**

The online version contains supplementary material available at 10.1007/s11695-021-05620-6.

## Introduction

The goal of this study was to compare existing methods for laparoscopic bowel length measurement (LBLM) with a novel computer-assisted 3D bowel measurement system (BMS) [1, 2].

Because of its numerous benefits for the patient, laparoscopic surgery has become the standard approach for a number of surgical procedures [3–5]. However, laparoscopy also poses relevant drawbacks, such as hindered instrument motion, loss of haptic feedback, and reduced depth perception [6]. Robotics and 3D-imaging technology have been proposed — and partially implemented — as a means of overcoming these problems [7], but no clinical standard for facilitating objective length or volume measurements laparoscopically exists. This aspect can be of significant clinical importance, e.g., when determining the extent of bowel resection during laparoscopic treatment of Crohn’s disease [8] or when ensuring adequate resection margins during laparoscopic treatment of colorectal cancer [9]. Bowel lengths also have to be measured for surgical reconstruction in urology, for example, to achieve optimal bladder size during the intraperitoneal creation of a neobladder after cystectomy [10] or in bariatric surgery during laparoscopic Roux-en-Y gastric bypass surgery (LRYGB) or biliopancreatic diversion with duodenal switch [11]. Determining the correct limb length in bariatric procedures is relevant for treatment success [12–15]. However, an online survey of practicing surgeons in the American Society for Bariatric Surgery revealed that only 53% of bariatric surgeons use a formally objective method (open grasper, premeasured umbilical tape, or suture) to measure the lengths of the constructed bowel limbs [16]. So far, methods for laparoscopic bowel length measurement (LBLM) have not been sufficiently assessed in clinical trials [17, 18], and there is no evidence for the superiority of one method of LBLM over the others [19].

This study thus aims to answer the research questions (1) whether a novel computer-assisted 3D measurement system is superior to previous LBLM methods and (2) how different LBLM methods compare regarding accuracy, measurement time, and the number of bowel grasps as a measure for the risk of bowel lesions.

## Materials and Methods

### Methods for Laparoscopic Bowel Length Measurement

**Fig. 1:**
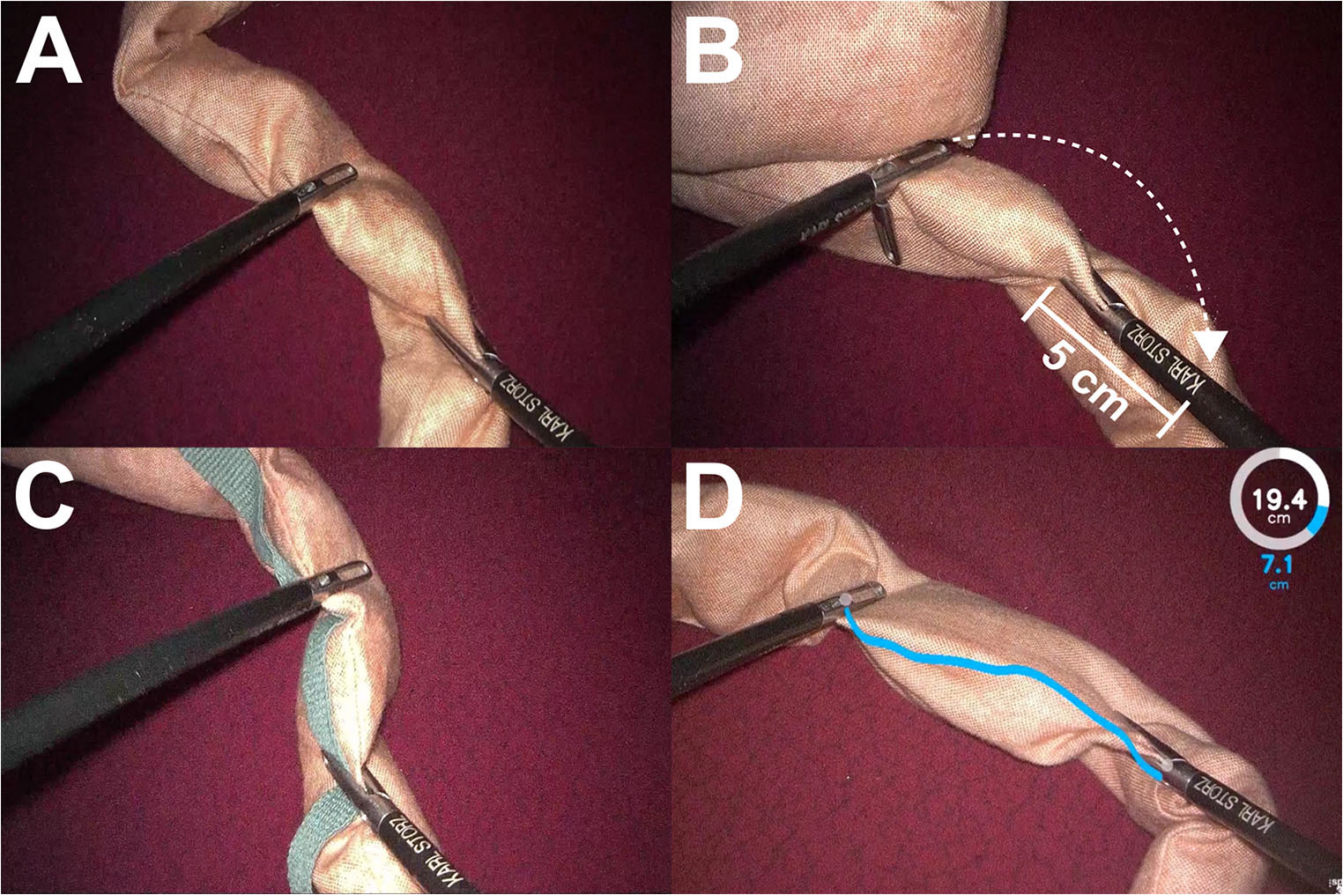
Methods for laparoscopic bowel length measurement (LBLM). **A** Visual judgment: bowel length is measured by mere visual estimation without a measurement tool. **B** Use of instrument markings: bowel is measured in 5 cm increment marks (letter “K” = 5 cm distance from instrument tip) on laparoscopic instruments. **C** Use of premeasured tape: bowel is handled along with premeasured tape of a specific length (here 35 cm). **D** Use of computer-assisted 3D bowel measurement system: bowel length is measured by a computer after pressing a foot switch and displayed to the surgeon in augmented reality as a measurement line between the instruments (blue line), as well as in a circle displaying both partial (blue) and total (white) measurements

A thorough literature search for articles describing LRYGB surgery and LBLM was performed. Out of the LBLM methods most commonly mentioned in the literature [13, 16–18, 20–27], visual judgment (VJ, Fig.1A), use of instrument markings (IM, Fig.1B), and use of premeasured tape (PT, Fig.1C) were selected. These methods were tested against the novel self-developed computer-assisted 3D bowel measurement system BMS (Fig.1D). In accordance to the survey performed by Madan et al. [16], we defined IM, PT, and BMS as formal LBLM methods because they use a measurement tool and compared these to the non-formal method VJ as a control.

#### Visual Judgment

VJ was defined as the laparoscopic measurement of a bowel length by mere subjective visual estimation of the operating surgeon without the use of a measurement tool (Fig. 1A).

#### Use of Instrument Markings

During IM, the surgeon measures bowel length with the help of measurement markings on the laparoscopic instruments (Fig. 1B). IM has been evaluated in a phantom model by Isreb et al. [17] and no difference in accuracy was found when using increments of 5cm or 10cm. Other studies also used 5cm [20], 10cm [21], both 5 and 10cm [22], or did not mention the increments used [13]. Thus there appears to be no clinically preferred increment length for IM. In this study, we chose 5cm for the sake of convenience.

#### Use of Premeasured Tape

When performing bowel measurement with premeasured tape or thread, the tape is used to estimate bowel length as the bowel is passed over it (Fig. 1C). Jackson et al. [18] evaluated PT in a phantom model with a group of medical students, residents, and surgical attendings. The study protocol included two LBLMs of 150cm on a 500cm rope with a 10-cm piece of thread [18]. A literature search yielded five clinical articles mentioning the use of PT [23–27] and one article mentioning the use of a premeasured silk thread for LBLM [28]. Since LBLM with a 10-cm thread requires the surgeon to grasp the bowel multiple times, thus hampering laparoscopic handling, we chose a 35-cm long premeasured umbilical tape for this study.

#### Use of Bowel Measurement System

This study utilized a newly developed computer-assisted 3D bowel measurement system (BMS) that has been described in full detail both from a surgical [1] and an engineering perspective [2]. In these studies, BMS proved both feasible and accurate in experimental as well as clinical settings [1]. In BMS, the surgeon holds a length of bowel with two laparoscopic graspers in front of the laparoscopic camera. A measurement command is given by pressing the left pedal of a foot switch and the measurement result is displayed via augmented reality. BMS adds each individual measurement to the total distance measured. Both the single measurements and the total distance are displayed via digits and a measurement circle (Fig. 1D). The desired total bowel length can be chosen beforehand, and when the full length is reached, a cross is displayed on the bowel.

### Study Design

All procedures performed were in accordance with the ethical standards of the institutional and/or national research committee and with the 1964 Helsinki Declaration and its later amendments or comparable ethical standards. After consultation of our institutional review board, we decided that a formal ethics approval was not necessary, because no patients were involved. To account for data privacy regulations, informed consent was obtained from all individual participants included in the study.

**Fig. 2:**
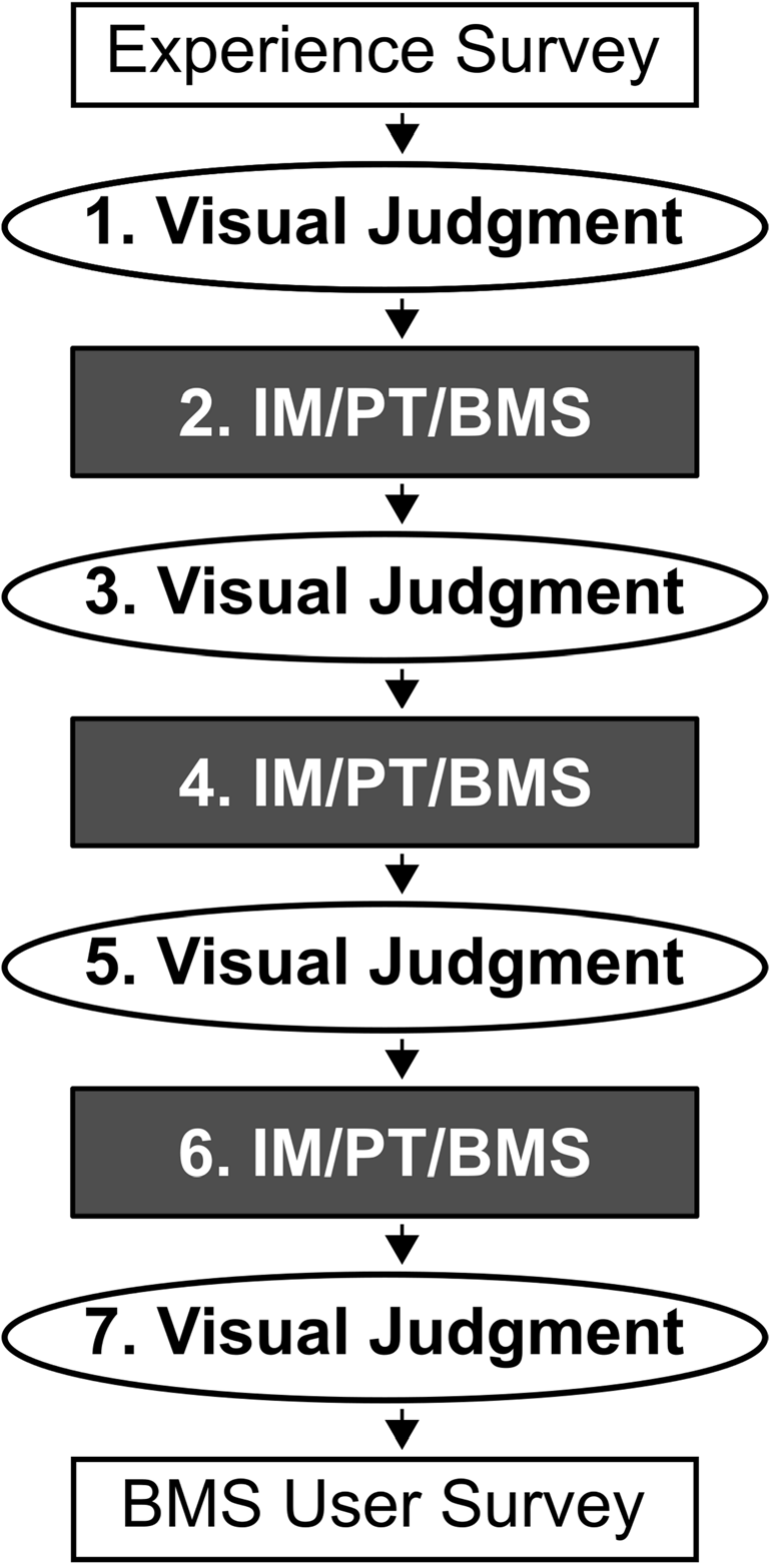
Study protocol. First, all participants completed an experience survey. Next, seven measurements were performed. Visual judgement was performed four times as the first, third, fifth, and seventh measurement for control of the learning curve. Measurements with use of instrument markings (IM), use of premeasured tape (PT), and computer-assisted 3D bowel measurement system (BMS) were performed once each following a randomized crossover design as the second, fourth, or sixth measurement in order to minimize confounding learning effects. Finally, a BMS user survey was completed

The study consisted of a total of seven measurements for each participant (Fig.2). The non-formal LBLM method VJ was performed four times as the first, third, fifth, and seventh measurement for control of the learning curve. The three formal LBLM methods, IM, PT, and BMS, were performed once each following a randomized crossover design as the second, fourth, or sixth measurement in order to minimize confounding learning effects. Because BMS requires a 3D laparoscope, all measurements were undertaken using a 3D laparoscope to avoid a 2D/3D bias, especially for VJ.

A target length of 70cm was chosen since it represents the length measured for the biliopancreatic limb in LRYGB at the Heidelberg Center for Diabetes and Obesity [29]. Before each measurement, participants watched an instruction video demonstrating instructions for the four different LBLM methods (Supplemental Digital Content 1) and were given time to get acquainted with each method. In order to avoid unnecessary bowel grasps, the video also instructed participants to measure the bowel along the seam using a hand-over-hand approach (grasping the next bowel stretch by readjusting only one of the two laparoscopic instruments). Moreover, following the seam prevented any twisting of the bowel that might occur due to an absence of mesentery. The instructor marked the start and end points of each measurement with pins and obtained ground truth manually with a tape measure after the measurement to calculate accuracy. Furthermore, measurement time and number of bowel grasps as a measure for the risk of bowel lesions were also recorded. Participants were blinded to the resulting bowel lengths they measured to minimize confounders resulting from learning effects.


Supplemental Video:Bowel Measurement Instructions.mp4 Video demonstrating instructions for the four different methods for laparoscopic bowel length measurement. (MP4 97476 kb)

In addition, all participants anonymously completed a survey after finishing the seven measurements (survey software: iPad Form Maker, Isoperla Ltd., Bath, UK). The survey evaluated user experience with BMS and comprised six questions with a 5-point Likert scale and two free text questions.

### Experimental Setup

**Fig. 3:**
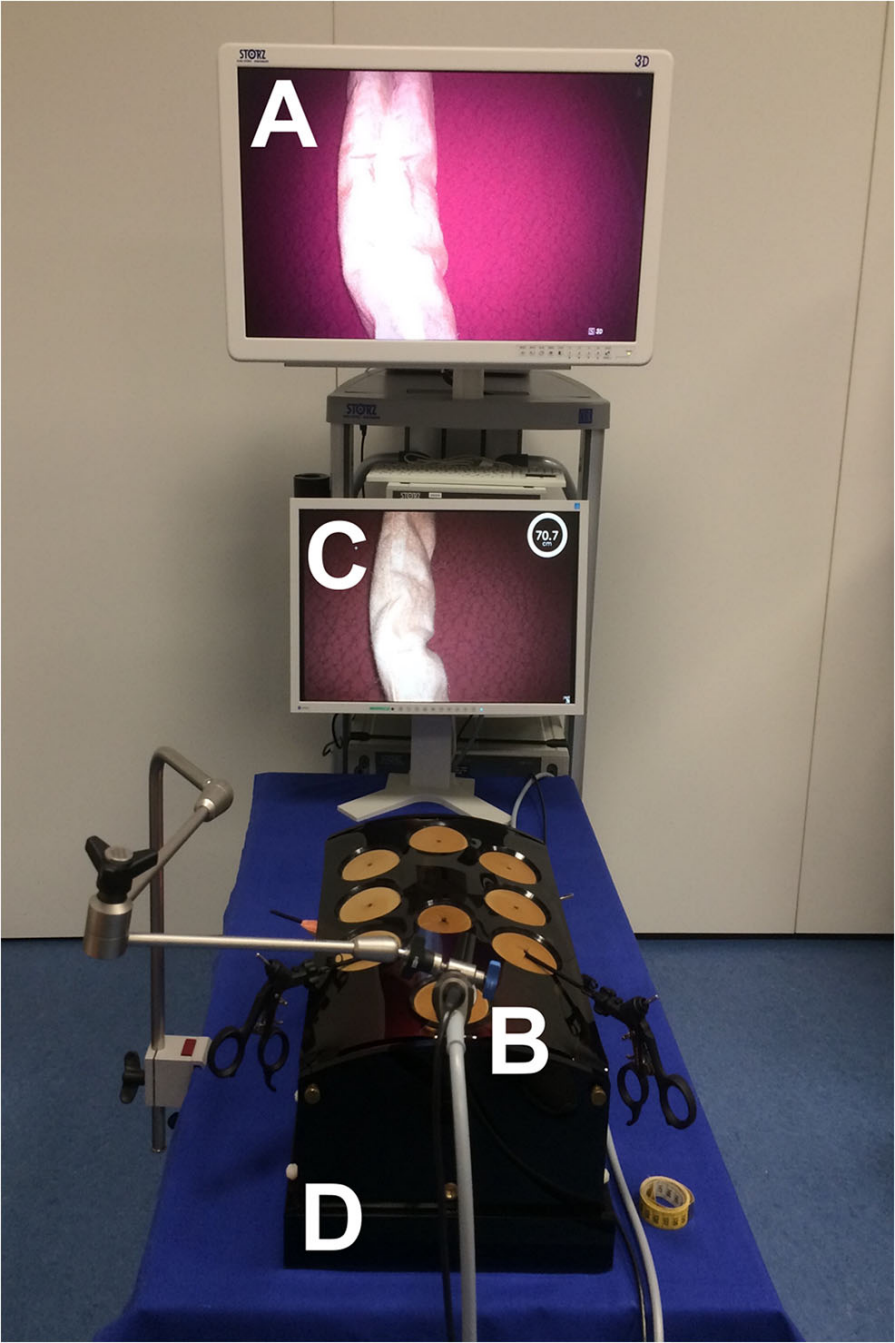
Experimental setup for the study. A 3D Monitor (**A**) shows the live image provided by the 3D laparoscope (**B**) while an LCD computer monitor (**C**) displays a 2D image with the augmented reality provided by the computer-assisted 3D bowel measurement system. All measurements were performed on bowel phantoms within a standard box trainer (**D**)

A standard box trainer model with a fixed 3D TIPCAM®1 HD laparoscope (Karl Storz GmbH & Co. KG, Tuttlingen, Germany) with a mounted camera head and a 30° optical system were used (Fig. 3). Images from the 3D laparoscope were viewed with 3D glasses on a 32" Medical 3D Full HD LCD Monitor (EJ-MDA32E-K, Panasonic Corporation, Kadoma, Japan). All measurements were performed using a xenon light source (Karl Storz GmbH & Co. KG, Tuttlingen, Germany) with 40% light intensity, a 60° camera angle, and a distance of 10cm from camera tip to bowel. Laparoscopic bowel graspers were used to handle the bowel phantoms. The bowel phantoms were crafted from cotton specifically for this study. Each had a length of 100cm, a diameter of 2.5cm, and was stuffed with an amount of cotton wool optimized for laparoscopic handling. A wooden board covered with a red and black pattered cloth provided a background image for simulating intra-abdominal measurements.

To run BMS, a personal computer (Intel Core i7-2700K CPU, GeForce GTX 650Ti GPU and 16GB RAM) with a video capture card (DVI2PCIe Duo capture card, Epiphan Systems Inc., Palo Alto, USA) and a foot switch (USB Foot Switch 2 Double, Scythe Co. Ltd, Tokyo, Japan) were used. The augmented reality user interface was displayed on a conventional 2D monitor.

### Statistical Analysis

Data were collected using the Microsoft Excel 2010 (Microsoft Corporation; Redmond, WA, USA). Statistical analysis and graph plots were performed with SPSS software (IBM, Armonk, NY) and R [30] with the integrated developer environment RStudio (RStudio Inc., Boston, MA, USA). Endpoints were accuracy, measurement time, and number of bowel grasps. As a further measurement of accuracy, relative error was calculated as the percent of difference between length measured with the respective LBLM method (70cm) and ground truth in cm, divided by ground truth in cm. Measurement time was logged as time in seconds needed for measuring 70cm. The number of bowel grasps needed to measure 70cm was also recorded. We chose the number of bowel grasps as a surrogate parameter for traumatic impact on bowel as extensive bowel handling increases the risk of bowel laceration in laparoscopic surgery [31]. Mean results for LBLM with VJ were used as a control for comparison with the three formal LBLM methods. Accuracy, measurement time, and traumatic impact were compared among LBLM methods using two-tailed paired *t* tests and one-way ANOVA. The learning curve with VJ was analyzed with one-way repeated measures ANOVA. Statistical significance was defined as *p*<0.05. BMS user survey data were analyzed using descriptive statistics.

## Results

In total, there were 63 participants performing the measurements in the study (21 surgical residents, 30 medical students, and 12 non-medical test persons).

**Fig. 4:**
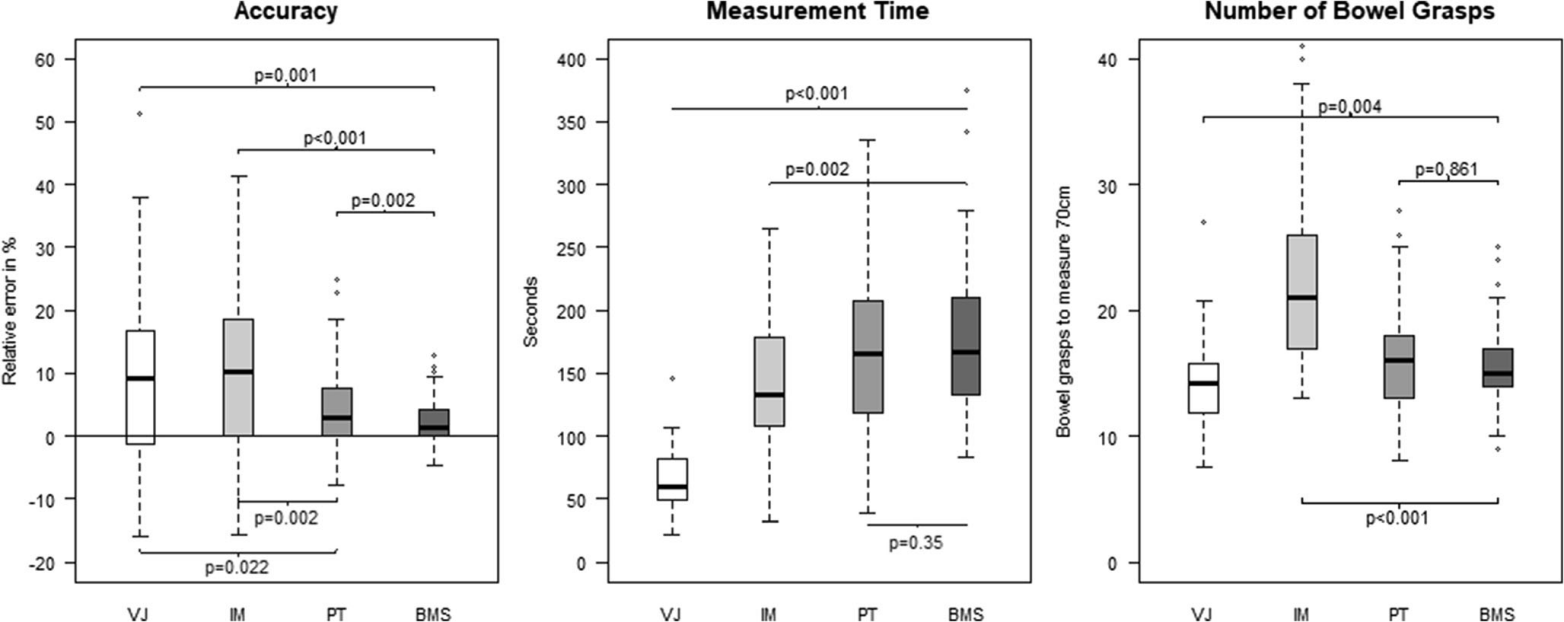
Study results. Box plots for accuracy calculated as relative error, measurement time in seconds, and number of bowel grasps as a measure for the risk of bowel lesions. Each measurement consisted of a length of 70 cm of phantom bowel. Results are displayed for laparoscopic bowel length measurement via visual judgment (VJ), use of instrument markings (IM), use of premeasured tape (PT), and use of computer-assisted 3D bowel measurement system (BMS)

The accuracy of LBLM was higher with BMS than with PT, IM, or VJ and higher with PT than with VJ or IM (Fig. 4). In absolute numbers, the measured length was 68.6±2.5cm (BMS, mean ± standard deviation), 67.4±4.2cm (PT), 64.2±8.4cm (IM), and 65.4±8.0cm (VJ) for the aim of measuring 70 cm. Regarding the learning curve, there was a trend toward VJ decreasing accuracy when comparing the first (8.0±26.8%), second (10.0±20.1%), third (13.6±19.5%), and fourth (11.0±16.9%) measurements, yet no significant learning curve could be observed (Wilks-Lambda 0.938, *p*=0.291). Subgroup analysis showed a higher accuracy for medical students (1.7±4.5%) than for residents (8.0%±8.0%, *p*=0.002) with PT and a higher accuracy for non-medicals (−0.2%±2.1%) than for residents (3.6%±4.7%, *p*=0.014) with BMS.

Measurement time was similar for BMS and PT, but shorter with VJ and IM (Fig. 4). A learning curve for VJ was observed (Wilks-Lambda 0.927, *p*<0.001); i.e., participants got progressively faster when comparing the first (80.8±42.2 s), second (63.8±30.5 s), third (55.7±26.1 s), and fourth (55.9±20.3s) measurements with VJ. Residents performed LBLM with VJ (52.5±22.0 s) faster than students (71.8±24.8 s, *p*=0.013). They also measured faster with IM (110.8±51.4s) than non-medicals (156.0±46.0s, *p*=0.049) and students (164.3±51.5s, *p*=0.001). LBLM with PT was faster when performed by residents (125.4±60.0 s) than by non-medicals (199.4±46.5 s, *p*=0.002) and students (182.6±61.7 s, *p*=0.003). No difference in measurement time between residents, students, and non-medical test persons was found for LBLM with BMS.

Number of bowel grasps per measurement of 70cm phantom bowel compared to BMS was similar with PT, but higher with IM and lower with VJ (Fig.4). Thus, all formal LBLM methods required more bowel grasps than VJ (IM: *p*<0.001, PT: *p*=0.003, BMS: *p*=0.004). No significant difference in number of bowel grasps needed to measure 70 cm between residents, students, and non-medical test persons was found. Analysis of the learning curve with VJ showed no difference for number of bowel grasps when comparing the first (14.3±4.5), second (14.3±4.3), third (13.7±3.7), and fourth (14.1±3.5) measurements (Wilks-Lambda 0.953, *p*=0.422).

**Fig. 5:**
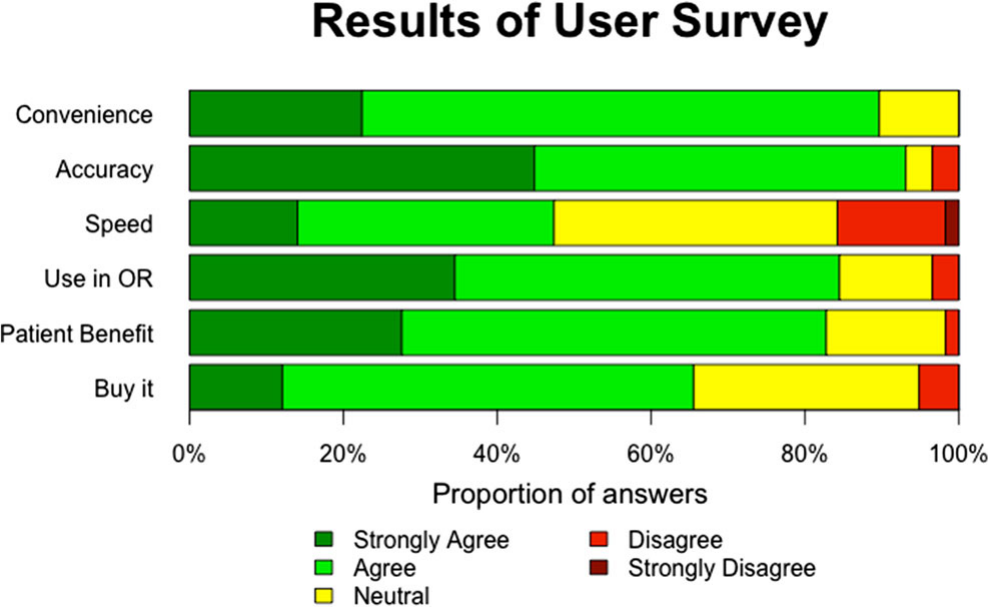
Results of user survey for computer-assisted 3D bowel measurement system (BMS). The statements users had to respond to on a 5-point Likert scale were as follows: (1) BMS is convenient, (2) BMS improves the accuracy of my bowel measurement, (3) BMS speeds up my workflow, (4) I would use BMS in the OR, (5) I believe my patient would benefit from BMS, and (6) If BMS was a product, I would buy it

The user survey revealed high overall satisfaction with BMS, especially regarding convenience, accuracy, and potential patient benefit. While responses regarding whether or not BMS might speed up workflow were divided, most participants stated that they would use BMS in the OR (Fig.5).

The most common suggestions for improvement (free-text question 1) were integration of two screens into one main screen (*n*=10, Fig.3 A and C), a different way to give measurement commands than via a USB foot switch, e.g., voice command or automatic measurement without an explicit command (*n*=5), more stable instrument detection (*n*=5), measurements in all angles and directions (*n*=4), and faster measurements (*n*=3).

When asked to evaluate BMS in a few words (free-text question 2), most answers could be subsumed under the categories: “user friendly” (*n*=16), “safer measurements” (*n*=10), “amazing” (*n*=7), “accurate method“ (*n*=5), “efficient” (*n*=4), “self-reassuring” (*n*=3), “takes training” (*n*=3), and “good for beginners” (*n*=3).

## Discussion

This study compared the novel computer-assisted 3D measurement system BMS to three different methods of laparoscopic bowel length measurement in a large study on a bowel phantom. We found that BMS shows great potential for more reliable LBLM. Also, PT had higher accuracy than VJ and IM, and lower number of bowel grasps than IM. Thus, until BMS is available in clinical routine, PT should be preferred for LBLM. Previous studies investigated the use of different methods for LBLM in preclinical models. Isreb et al. investigated the use of IM vs. VJ [17], and Jackson et al. investigated the use of PT vs. VJ [18]. In contrast, our study presents a comparison of the novel measurement method of computer-assisted 3D bowel length measurements to all three previously investigated methods. Thus, the most important contributions of this study for practicing metabolic surgeons are both, a structured comparison of commonly existing methods LBLM with recommendations for the use in the operating room, as well as an outlook what may improve the state of the art with computer-assistance.

### Computer-Assisted 3D Bowel Length Measurement

BMS as a method to perform LBLM turned out to be very accurate and reliable in our phantom study. The accuracy of BMS was even superior to all three methods that are currently available in the clinical routine. Moreover, BMS produced a very low variance in measurement, especially when compared to VJ and IM. The number of bowel grasps as a measure for the risk of bowel lesions was also low and comparable to VJ or PT. Furthermore, with BMS, the surgeon did not need to count measurements and could measure individual stretches of bowel without having to introduce another object (premeasured tape) into the patient’s abdomen which means less foreign body risks.

In terms of clinical application, BMS could be used in metabolic surgery, at least for procedures with a higher risk of malabsorption such as biliopancreatic diversion (BPD), single anastomosis duodeno-ileal bypass (SADI), or one anastomosis gastric bypass (OAGB). Furthermore, BMS’ accurate LBLM could help with the creation of an optimal neobladder size in robotic cystectomy or quantify bowel resection length in the surgical treatment of Crohn disease.

However, LBLM with BMS was more time-consuming than the other methods, a fact which could be explained by the experimental setup’s sub-optimal user interface: participants had to view the measurement result on a separate screen (Fig.3C). Accordingly, screen integration and changing the command interface were the most common suggestions for improvement in the user survey.

### Structured Comparison of Commonly Used Methods

PT was the most reliable and accurate LBLM method of those currently available in the clinic, offering the major advantage that participants could pass the tape alongside the bowel in long increments without having to count and sum up each measurement. Laparoscopic handling would likely be further eased in vivo by an adhesive effect between small bowel and umbilical tape. However, the surgeon is obliged to introduce a foreign body into the patient’s abdomen and can only measure distances set by the length of the premeasured tape and by the available space in the surgical field. Jackson et al. performed a trial that compared LBLM with a premeasured suture of 10cm to LBLM with VJ [18] and found the use of PT increased precision without effecting operative time or procedural flow. While increase in precision was consistent in the present study, we found that measurement time more than doubled. However, the increase in measurement time was accompanied by higher accuracy.

IM is the most common formal LBLM method used by bariatric surgeons during LRYGB surgery [16]. However, our study showed that IM yielded the same low accuracy as VJ, in addition to posing a twofold increase in measurement time and requiring higher number of bowel grasps. The increased number of bowel grasps is of particular clinical interest as extensive bowel handling increases the risk of bowel laceration in laparoscopic surgery [31]. These findings conflict with the results from a study by Isreb at al. [17], in which participants measured significantly more accurately when using IM instead of VJ. In that study, 22 participants measured a distance of 150cm on a piece of string with VJ and then with IM. This order of LBLM might have led to a training affect for participants and therefore better results with IM. In our study, we controlled that bias by randomizing the order and repeating measurement with VJ as a control. Also, Isreb et al. did not investigate the additional number of bowel grasps IM required for readjusting bowel to match the increment length on the marked instrument.

In the present study, VJ was a fast LBLM method, with a low number of bowel grasps. However, VJ was also inaccurate (with a relative error of 8.7%) and unreliable (with a standard deviation of 13.7%). Also, in a previous study showing the first human use of BMS, BMS measured 38.3cm for a target length of 60cm that was estimated with VJ (−36.2% relative error). In the same study, the relative error of BMS was always positive and well below +10% in phantom, ex vivo porcine and in vivo porcine experiments [1]. Moreover, participants showed a decrease in accuracy over the four measurements with VJ that correlated with a simultaneous reduction in measurement time. However, this could also be explained by a decrease in motivation for the study.

Overall participants showed a trend to underestimate the target length of 70cm independent of the LBLM method, with a positive mean relative error for all four methods. This finding is consistent with those of Jackson et al. [18] and Isreb et al. [17]. Magnification of the surgical field by the laparoscope likely contributes to this underestimation. In addition, participants tend to perform LBLM in a zigzag pattern along the bowel surface, since it is difficult to measure a flexible tubing structure in a straight line. In order to minimize this effect in our study, participants were instructed to measure along the seam of the bowel phantom.

### Study Limitations and Future Research

As our target length of 70 cm differed from the length chosen for previous studies (150cm) [17, 18], our total deviation in cm from the target length was also smaller. Choosing a target length longer than 70cm would probably even have increased the effects we found. Nevertheless, to account for this smaller deviation, we defined accuracy as relative error. This normalization allowed for a better comparison of LBLM methods independent from the chosen target length.

Bowel phantoms made from cotton were chosen for the present study to mimic laparoscopic bowel handling. While this method constitutes a more realistic bowel model than a piece of thread [17] or rope [18], it still does not fully account for the tonicity and flexibility of human bowel. In the face of the current controversy regarding whether bowel tonicity does [32] or does not [27] affect small bowel length measurement, further studies should compare LBLM methods with regard to accuracy, measurement time, and traumatic impact in vivo, for example in animal studies. Here, it should also be investigated to which extend small bowel measurement depends on tissue stretching and bowel tonicity to account for intraindividual measurement differences in the future.

Furthermore, our study included a reasonable number of surgical residents, medical students, and non-medical test persons, but no expert bariatric surgeons. We chose this study design as an exploratory study to estimate the potential clinical impact of the novel system BMS. Here, it may even be beneficial to start with less experienced users to find the real difference between BMS and the other methods, without an experts’ experience compensating the shortcomings of inferior methods. Future studies should investigate clinical translation, i.e., whether our results can be reproduced with expert bariatric surgeons performing in vivo measurements which would also account for the problems of tonicity (see above).

Since BMS requires a 3D camera, all LBLM methods were compared employing 3D rather than 2D display. The effect of 3D vision on accuracy, number of bowel grasps, and speed of LBLM methods has not yet been evaluated. Previous studies have shown that 3D vision results in better depth perception [33], faster laparoscopic task completion [34], and faster learning of laparoscopic skills in inexperienced test persons [35]. Similarly, participants in our study might have had better results because our study used 3D, instead of 2D, display. Further studies investigating the influence of 3D display on LBLM are therefore needed. These benefits, however, have to be weighed against potential side effects of 3D laparoscopy (e.g., headache) that may be caused if setup is not optimal according to an EAES consensus on 3D in laparoscopy [36] and have been described by some, but not all studies are included in a recent systematic review on 3D in urological laparoscopy [37].

Finally, BMS is a novel system and this study was the first test of the system in a larger cohort of participants. To refine the system for future studies based on suggestions by the participants, two measurement screens should be integrated into one screen and the computer algorithms should be refined to allow for improved measurements, i.e., more robust instrument detection, measurement from different angles, and faster measurement.

## Conclusion

In this study with a bowel model, BMS shows great potential for clinical use. Its high accuracy and reliability as well as its low traumatic impact could enable objective LBLM in clinical practice and therefore help to standardize laparoscopic procedures such as LRYGB surgery. However, additional technical refinements and studies are needed for clinical validation.

As long as BMS is not available in the clinical routine, based on this model, study using an objective method for LBLM is recommended at least for procedures with higher risk of malabsorption (BPD, SADI, OAGB), because of the limited accuracy and high variance of VJ. As a method of choice, we recommend PT, due to its accuracy and requisite low number of bowel grasps. These recommendations may also be extrapolated to other fields of surgery such as the creation of an optimal neobladder size in robotic cystectomy or quantify bowel resection length in the surgical treatment of Crohn disease.
